# The impact of low-carbon city pilot policy on urban green technology innovation: Based on government and public perspectives

**DOI:** 10.1371/journal.pone.0306425

**Published:** 2024-07-10

**Authors:** Qingjie Pan, Shouguo Zhao

**Affiliations:** School of Economics and Management, Northwest University, Xi’an, China; Inner Mongolia University, CHINA

## Abstract

Global climate change has caused a series of environmental problems, green technology innovation is necessitating strategic responses, but the impact of low-carbon city pilot policy on urban green technology innovation is unclear. Based on panel data from 285 Chinese cities during 2005–2022, this study employs the Difference in Difference method to examine the impact of low-carbon city policy on urban green technology innovation. The results show that (1) The low-carbon city pilot policy promotes urban green technology innovation. (2) The low-carbon city pilot policy promotes urban green technology innovation through government green input and public engagement. (3) New infrastructure enhances the impact of low-carbon city pilot policy on quantity of green technology innovation. (4) Compared with the Yangtze River Economic Belt, the low-carbon city policy has a greater influence on urban green technology innovation in the Yellow River Basin.The findings provide policy insights for the construction of low-carbon pilot cities.

## 1. Introduction and literature review

Global climate change has caused a series of environmental problems. How to solve environmental problems has become the primary consideration of sustainable development. Green technology innovation can achieve sustainable development by enhancing energy efficiency and reducing carbon emissions, which can mitigate climate change [1]. However, green technology innovation presents negative external and high-risk characteristics that, if it is not able to form a competitive advantage, hinder its development. China’s low-carbon city pilot policy emerges as a pivotal framework providing policy direction for the advancement of green technology innovation. Therefore, can the low-carbon city pilot policy effectively stimulate the development of green technology innovation? What is the role mechanism of the low-carbon city pilot policy to influence green technology innovation? This study is designed to explore the impact of low-carbon city pilot policy on urban green technology innovation by employing theoretical explanations and empirical tests.

The literature on green innovation and the low-carbon city pilot policy is reviewed in this paper. First, according to the “Porter hypothesis”, environment regulation influences green technology innovation [2,3]. The “Porter Hypothesis” typically serves as the foundation for literature on the effects of environmental regulation on green technology innovation, including the weak “Porter Hypothesis” and strong “Porter Hypothesis”. The weak “Porter Hypothesis” asserts that environmental regulation could stimulate innovation inputs in the longer term, however, which do not immediately disclose innovation outputs and competitiveness. For testing the weak “Porter Hypothesis”, Milani S. found that increased environmental regulation can force enterprises to increase R&D spending [4]. The strong “Porter Hypothesis” asserts that environmental regulation can stimulate enterprise technological innovation, enhance enterprise market competitiveness, and offset the additional costs arising from environmental regulation. For testing the strong “Porter Hypothesis”, some studies discovered that environmental policy can promote productivity in specific industries [5,6], and Chen et al. found that pilot policy forms the spatial spill effect [7]. Based on green technology innovation is the core competitiveness of sustainable development. El-Kassar et al. divided green technology innovation into green process innovation and green product innovation [8]. Zhang et al. divided green technology innovation into real green technology innovation and strategic green technology innovation, and found that strategic green technology innovation seems more sensitive to environmental regulation. Additionally, high fiscal subsidies and industrial agglomeration are conducive to helping the environmental regulation induce real green technology innovation [9]. From the perspective of government behaviors, Guo et. al found that direct government funding and tax incentives can promote green technology innovation, but the promotion of government tax incentives to green technology innovation is not significant [10]. From the perspective of policy types, Environmental regulation mainly includes command-based environmental regulation, market-based environmental regulation and voluntary environmental regulation. Zhang et al. investigated the impact of three environmental regulation on green innovation and discovered that market-based and voluntary environmental regulation are more effective in promoting green innovation than command-based environmental regulation [11]. Meanwhile, Fang et al. found that green finance and market-based environmental regulation can promote regional green technology innovation, while command-based environmental regulation inhibit regional green technology innovation [12]. Another part of the study shows that command-based, market-based and voluntary environmental regulation have the opposite effect. Wang et al. found that command-based and market-based environmental regulation could stimulate enterprises to carry out green technology innovation, and the promotion effect of green utility model patents is slightly higher than that of green invention patents. voluntary regulation inhibits enterprise green technology innovation [13]. Furthermore, other studies found the factors influencing green technology innovation, which include the green credit [14], digital finance [15],environmental information disclosure [16].

Second, Existing studies mainly focus on the consequences of the low-carbon city pilot policy from two main directions. The Difference in Difference (DID) method is also used by most scholars to evaluate the impact of the low-carbon city pilot policy. From the perspective of pollution reduction and green development. Shi et al. believed that the low-carbon city pilot policy can effectively promote urban pollution reduction and green development [17]. Huo et al. explored the intermediary mechanism and found that pilot cities reduced emissions by adjusting their industrial structure, promoting technological innovation of enterprises to increase their total-factor productivity, and stimulating green technology innovation [18]. Regarding the heterogeneity. Cheng et al. examined the effect of the low-carbon city pilot policy on green Gross Domestic Product at the city level and found that the low-carbon city pilot policy has an economy of scale effect and regional differences [19]. Liu et al. demonstrated that the low-carbon city pilot policy has effectively promoted the reduction of carbon dioxide emissions brought about by the growth of GDP per unit, and the policy effect of the pilot cities in eastern China is significant, while in western and central China is not. Energy structure, industrial structure, and innovation level have a significant impact on the effect of low-carbon city pilot policy [20]. Meanwhile, Fu et al. believed that the low-carbon city pilot policy’s influence on carbon reduction is not always immediate and that policy effects are more effective in the eastern region than the western region [21]. Additionally, Wen et al. found that the low-carbon city pilot policy has heterogeneous impacts on carbon emissions levels in different cities, with lower resource dependency, higher economic levels and more significant emission reductions in cities with high political status [22]. Pan et al. found that the low-carbon city pilot policy significantly promotes low-carbon innovation, and more significantly impacts innovation with higher carbon reduction potential and promotes more innovation in small-sized and medium-sized cities [23]. Regarding the spatial spillover effect, Yan et al. demonstrated that the implementation of the low-carbon city pilot policy effectively reduces pollution in China and that the low-carbon city pilot policy has spatial spillover effects [24]. Chen et al. found that the low-carbon city pilot policy has a siphon effect and increase the carbon intensity of neighboring regions [25]. Meanwhile, Chen et al. explored the spatial spill effect and found that pilot policies for low-carbon cities can simultaneously accelerate green innovation processes in local and surrounding cities and can better space spill effects in high-level cities, larger towns and eastern cities [26]. Other scholars suggested the opposite, Tian et al. looked at the impact of the low-carbon city pilot policy on urban green technology innovation, and found that the pilot policy not only did not have a positive effect on urban green technology innovation, but lowered the overall green technology innovation level of the city [27].

First, although there is a rich literature to test the “Porter hypothesis,” the more studies mainly examine environmental regulation, rather than the low-carbon city pilot policy. Second, although some literature has evaluated the consequences of the low-carbon city pilot policy, the selected assessment criteria have mostly focused on environmental pollution and green development. Third, the impact of low-carbon city pilot policy on green technology innovation is unclear, and existing studies have not explored the specific mechanisms from government and public perspectives.

This paper makes the following marginal contributions: (1) A useful addition to the literature on the implementation effect of low-carbon city pilot policy, this paper analyzes the pilot policy effects from the perspective of urban green technology innovation, and divides green technology innovation into the quantity and quality of green technology innovation. (2) This paper analyzes the impact mechanisms of low-carbon city pilot policy on urban green technology innovation from two perspectives: government green input and public engagement. (3) New infrastructure provides new opportunities to promote green technology innovation. Therefore, it is of practical significance to identify how the low-carbon city pilot policy affects green technology innovation under new infrastructure. This paper analyzes the moderated mechanism of new infrastructure. (4) The Yellow River Basin and the Yangtze River Economic Belt are important regional economies in China, this paper examines the heterogeneity of pilot policy. So that this study can capture the space effect of pilot policy more precisely.

## 2. Theoretical analysis and research hypotheses

### 2.1 The low-carbon city pilot policy and green innovation

Green technology innovation refers to product and technology that reduce environmental pollution [28,29]. Traditional technology innovation emphasizes the investment of R&D funds, and disregards the costs to environment and resource production during technology innovation process. Green technology innovation focuses on the consumption of ecological resource and environmental pollution in the production process, and emphasizes the importance of technological innovation for sustainable development of society [30,31].Green technology innovation has its own features because, in contrast to traditional technology innovation. First, green technology innovation has the double effects of green and innovation, and it cannot monopolize the results of environmental improvement brought by green technology innovation, presenting negative externality characteristics. Second, green technology innovation often has financing constraints, due to the substantial investment required and the lengthy development cycle associated with green technology innovation, so green innovation effect is uncertain, presenting high-risk characteristics [32]. The characteristics of green technology innovation affect the development of innovative green technologies of cities. The pilot cities have introduced low-carbon city pilot policy based on their industrial structure, resource endowment, and technological advantage. Low-carbon city pilot policy aims to make up for the costs of green technology innovation through the benefits of green technology innovation, constraining the negative externalities [33]. Additionally, the development of green technology receives more funding from pilot cities in order to realize the green development. Second, the low-carbon city pilot policy is a comprehensive environmental regulatory policy, mainly includes command-based, market-based and voluntary environmental regulation [34]. Command-based environmental regulation is characterized by clear objectives, easy operation, and easy regulation, and usually, the government achieves the policy objectives through strong external interventions to influence urban green technology innovation delayed by negative externalities. Market-based environmental regulation serves as a more flexible regulatory tool, it is based on the market operation mechanism, through the influence of cost-benefit conduction policy guidance, resulting in the "innovation compensation" effect, and promoting the development of urban green technology innovation. Voluntary environmental regulation is based on educational activities to raise public awareness and participation in low-carbon development. Based on this, this paper proposes the following hypothesis:

Hypothesis H1: The low-carbon city pilot policy promotes urban green technology innovation.

### 2.2 Intermediary mechanism analysis

The government, as the principal entity responsible for implementing low-carbon city pilot policy, actively plays a guiding role in the program’s execution. Government green input is an essential instrument for the government to actively participate in green development. Therefore, government green input affects the development of innovative green technologies of cities from different aspects. In the administrative aspect, government green input demonstrates that pilot cities implement policies conducive to low-carbon development. Specifically, pilot cities increase the construction of low-carbon infrastructure, and provide infrastructure support for green technology innovation. In the economic aspect, government green input demonstrates that the government is conducive to reducing the city’s burden on social low-carbon governance. Specifically, pilot cities provide financial subsidies and tax incentives to encourage cities to adopt low-carbon technologies and reduce carbon dioxide output [35]. In the legal aspect, government green input demonstrates that the government improves urban regulations and standards. Specifically, pilot cities provide legal reference for promoting low-carbon policies and maintain the external environment for continuous green technology innovation. Based on this, this paper proposes the following hypothesis:

Hypothesis H2: The low-carbon city pilot policy promotes urban green technology innovation through government green input.

The Public, both as the participant and as the beneficiary in the low-carbon city pilot policy, has influenced the development of urban green technology innovation in two aspects. As the main participant, public engagement improves low-carbon literacy. The public pays more attention to the environmental quality of cities, and puts higher demand on urban energy conservation and emission reduction, Therefore, public engagement creates effective external monitoring mechanism that forces high-pollution cities to step up green technology innovation. As the principal beneficiary, public engagement increases awareness of the value of low-carbon property, increases demand for low carbon product, and uses from the benefit and improvement of green technology innovation [36,37]. The theory of “Demand leads to Innovation” proposes inventions are driven by knowledge or by demand. According to the above theory, technology innovation is a kind of market behavior. Public engagement affects the cost of green technology innovation, and green technology innovation cost include green innovation unit cost and market cost. In the green innovation units cost aspect. Green innovation is high investment. Public engagement shares the R&D cost, increases the expected level of R&D profit, and inspires urban green technology innovations. In the green innovation market cost aspect. Green innovation is often under-recognized. Innovators spend high costs on continuous market opening, in order to increase market awareness and consumer acceptance. Public engagement increases the market recognition of low-carbon products, reduces the cost of green technology innovation to open up the market, and inspires urban green technology innovations. Based on this, this paper proposes the following hypothesis:

Hypothesis H3: The low-carbon city pilot policy promotes urban green technology innovation through public engagement.

### 2.3 Moderated mechanism analysis

New infrastructure includes information infrastructure, convergence infrastructure, and innovation infrastructure. First, information infrastructure expands the production and trading space, achieves full-scale information tracking of production and transaction links, effectively utilizes large amounts of data information, enhances the ability to extract innovative elements from existing information, thus promoting urban green technology innovation. Second, convergence infrastructure facilitates the digital transformation of traditional industries. By optimizing the combination of factors, enhancing the process of innovation, and shortening the innovation cycle, convergence infrastructure effectively reduces the R&D cost, which makes it easy to alleviate the financing constraints in the urban green transformation. Third, innovation infrastructure provides basic support for green technology innovation, realizes the accumulation of green technology innovation.

New infrastructure effectively reduces information asymmetry, helps the government and the public to recognize green technology innovation. It helps the government to effectively select projects with stronger green technology innovation signal, and also helps the public choose products with strong green technology innovation signal. However, it should not be overlooked that information is not equal to knowledge, and knowledge is formed only after information is structured, processed and compiled. Different information recipients have different perceptions of information, different degrees of access to information, and different contents, so that knowledge is rooted in the recipient of information. Due to the limitation of expertise and specific practice, it is difficult for the government and public to judge the quality of green technology innovation, and pilot cities, in order to obtain more government green input and public engagement, tend to increase quantity of green technology innovation expressed by short development cycle. Based on this, this paper proposes the following hypothesis:

Hypothesis H4: New infrastructure enhances the impact of low-carbon city pilot policy on quantity of green technology innovation.

### 2.4 Heterogeneity analysis

**Fig 1:**
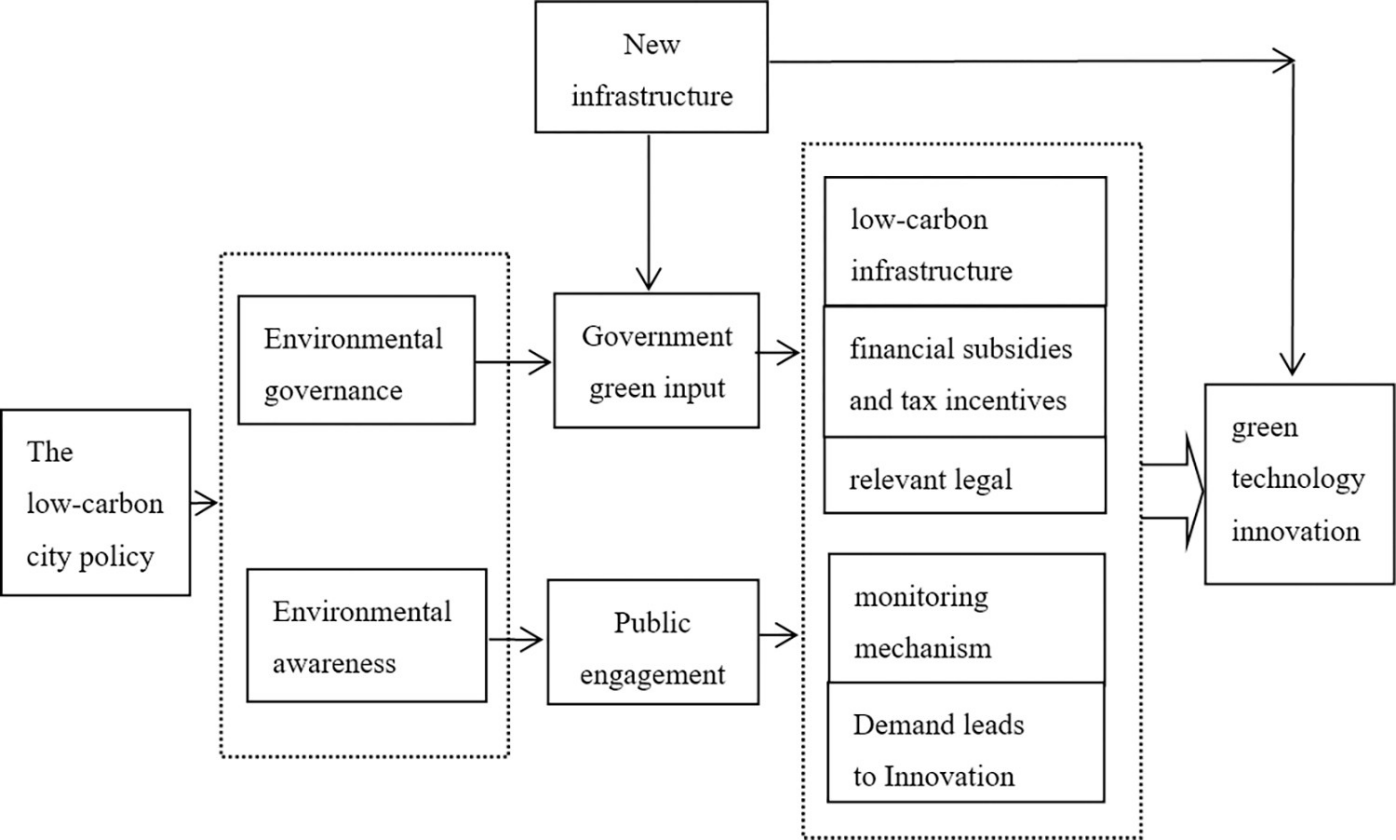
The impact of low-carbon city pilot policy on urban green technology innovation.

Basin economy belongs to sub-regional and trans-regional economy, which profoundly affects national economic development [38]. Yellow River Basin and the Yangtze River Economic Belt influence the effects of pilot policy for low-carbon cities from the geographical units of the basins. The agglomeration effect affects technology innovation [39]. Yellow River Basin and the Yangtze River Economic Belt present different patterns of agglomeration. The cities of Yellow River Basin are balanced distribution, forming two isolated skeleton networks with the city of Jinan and Qingdao as the core and the city of Zhengzhou and Xi’an as the core. The center cities of the Yangtze River Economic Belt are highly concentrated, forming a skeleton network with the city of Shanghai and Nanjing as the core. Different agglomeration modes form different agglomeration effects. the agglomeration effect of Yangtze River Economic Belt has cost comparative advantage for green technology innovation. Compared to the Yangtze River Economic Belt, Yellow River Basin has not yet formed innovation cost advantage. Government green input effectively reduces the cost of green technology innovation, significantly promotes green technology innovation in the Yellow River Basin. Additionally, Yangtze River Economic Belt has a high level in both pollution reduction and resource efficiency. Compared to the Yangtze River Economic Belt, Yellow River Basin is rich in minerals and natural resources, which has higher requirement for ecological protection in the development process, and it is urgent to improve the overall factor productivity and achieve green development. Public participation supervises the implementation of low-carbon city pilot policy, has a significant impact on green technology innovation in the Yellow River basin. Based on this, this paper proposes the following hypothesis:

Hypothesis H5: Compared with the Yangtze River Economic Belt, the low-carbon city policy has a greater influence on urban green technology innovation in the Yellow River Basin (See Fig 1).

## 3. Model construction and variables description

### 3.1 Model construction

The low-carbon city pilot policy leads to differences between pilot and non-pilot cities, before and after the implementation of the policy in the pilot cities. As a result, this study builds the Difference in Difference (DID) method to analyze the impact of low-carbon city pilot policy on green technology innovation. China’s low-carbon city pilot policy is carried out three batches, which means that the pilot time of each city is different, this study adopts the multi-period DID regression analysis. The specific model is as follows:

$$Y_{it}=\alpha +\beta D_{it}+\delta X_{it}+\mu_{i}+\upsilon_{t}+\varepsilon_{it}$$ (1)

In Eq (1), i represents city, i = 1,2,…,285. t represents year, t = 2005,2006,… . .2022. the explained variable Y_it_ represents green technology innovation in the “i” city, in the “t” year. the explanatory variable D_it_, D_it_ = Treat_i_ *Period_it_, is the treatment effect dummy variable, Treat_i_ is the treatment group dummy variable, in the treatment group the value is taken as 1, otherwise the value is taken as 0. Period_it_ is the treatment period dummy variable, in the treatment period the value is taken as 1, otherwise the value is taken as 0. the average treatment effect β indicates the difference between treatment group’s before and after changes and control group’s before and after changes, reflecting the influence of low-carbon city pilot policy on green technology innovation, the β coefficient is significantly positive, demonstrating that the low-carbon city pilot policy significantly improves the state of green technology innovation, and vice versa, the β coefficient is significantly negative, suggesting that the low-carbon city pilot policy has a detrimental effect on the state of green technology innovation. The control variables X_it_ represent the city-level control variables that change over time, including economic development, industrial structure, degree of openness, fiscal support, and human capital.

### 3.2 Variable description

This study is based on 285 cities in China from 2005 to 2022, and basic data are all from the Chinese Research Data Services (CNRDS). This study rejects samples with seriously missing data in the list. Additionally, some missing data are supplemented by interpolation.

Explained variable: green technology innovation. In this study, green technology innovation is measured along two dimensions, specifically the quantity of green technology innovation (Quantity) and the quality of green technology innovation (Quality). Based on existing research [40,41], The quantity of green technology innovation is measured by the total number of applied green patents, which includes the number of applied green inventions and applied green utility models in a given year. This indicator expressed by logarithm of total number adds 1 is a positive measure, where a higher value indicates greater quantity of green technology innovation, and vice versa. As patents represent stronger innovation ability and better reflect the quality of innovation activities [42], the quality of green technology innovation is measured by the proportion of applied green inventions to the total number of applied patents (applied inventions, applied utility models and applied appearance designs) in a given year. This indicator is also a positive measure, where a higher value indicates higher quality of green technology innovation, and vice versa.

Core Explanatory Variable: Low-carbon city pilot policy (Low-carbon). In this study, the core explanatory variable is a dummy variable for whether or not the 285 cities in China have launched low-carbon city pilot policy from 2005 to 2022, and the variable is taken as 1 for cities that implemented low-carbon city pilot policy, and 0 for other cases.

Based on previous studies [43,44], the control variables selected in this study include (1) Economic development (Pgdp), expressed by logarithm of GDP per capita. (2) Industrial structure (Industrial), measured by the proportion of secondary industry to GDP. (3) Degree of openness (Open), measured by the percentage of foreign direct investment to GDP. (4) Fiscal support (Fiscal), measured by the percentage of scientific and technological expenditure to public fiscal expenditure. (5) Human capital (Human), measured by the percentage of students in universities to household population at the end of the year.

See Table 1 for descriptive statistics of explained variable, core explanatory variable and control variables.

**Table 1:** Descriptive statistics of variables.

Variable	Observation	Mean	Std. Dev.	Min	Max
Quantity	5048	4.33737	1.86589	0	10.30075
Quality	5048	2.44156	1.77423	0	36.09756
Low-carbon	5048	0.24168	0.42814	0	1
Pgdp	5048	10.46826	0.76992	4.59512	13.05569
Industrial	5048	0.46318	0.11383	0.09	0.9097
Open	5048	1.50643	1.91674	0.00018	19.78282
Fiscal	5048	1.46738	1.61126	0.00002	20.68348
Human	5048	1.77214	2.29541	0.00592	14.89434

### 3.3 Low-carbon city characteristics

Low-carbon cities signify a transition towards more environmentally friendly resource allocation and achieving comprehensive low-carbon urban development. Low-carbon city pilot policy represents soft constraints, where specific low-carbon targets are not set at the central government, and the responsibility for advancing low-carbon initiatives is delegated to local governments. Therefore, examining the differences in the quantity and quality of green technology innovation between pilot and non-pilot cities can provide insights into the level of green innovation development in pilot cities. As shown in Table 2, the column (1) represents the quantity of green technology innovation in pilot cities, while the column (2) represents the quality of green technology innovation in pilot cities. The column (3) represents the quantity of green technology innovation in non-pilot cities, while the column (4) represents the quality of green technology innovation in non-pilot cities. The column (5) represents the proportion of quantity in pilot cities to quantity in non-pilot cities, while the column (6) represents the proportion of quality in pilot cities to quality in non-pilot cities. Overall, both the quantity and quality of green technology innovation have been improving over the years, with significant differences observed between pilot and non-pilot cities. Regarding the quantity of green technology innovation, pilot cities consistently maintain higher levels than non-pilot cities, with the lead gradually increasing from 1.451 to 1.781. Regarding the quality of green technology innovation, pilot cities consistently maintain higher levels than non-pilot cities, with the lead gradually increasing from 1.188 to 1.745.

**Table 2:** Level of green technology innovation in pilot and non-pilot cities.

Year	Pilot Cities		Non-pilot Cities		Proportions	
	Quantity1 (1)	Quality1 (2)	Quantity2 (3)	Quality2 (4)	Quantity1/Quantity2 (5)	Quality1/Quality2 (6)
2005	2.853	2.091	1.966	1.760	1.451	1.188
2006	3.148	2.347	2.110	2.010	1.492	1.168
2007	3.307	2.487	2.190	2.115	1.510	1.176
2008	3.506	2.761	2.225	2.118	1.576	1.304
2009	3.810	2.985	2.172	2.133	1.754	1.399
2010	4.075	3.251	2.426	2.146	1.680	1.515
2011	4.297	3.510	2.179	2.321	1.972	1.512
2012	4.651	3.851	2.537	2.235	1.833	1.723
2013	4.812	3.962	2.690	2.325	1.789	1.704
2014	5.068	4.252	2.858	2.531	1.773	1.680
2015	5.356	4.564	2.879	2.644	1.860	1.726
2016	5.616	4.712	2.970	2.623	1.891	1.796
2017	5.913	4.935	3.150	2.495	1.877	1.978
2018	6.059	5.071	3.285	2.764	1.844	1.835
2019	6.176	5.149	2.767	2.267	2.232	2.271
2020	6.394	5.354	2.529	2.164	2.528	2.474
2021	6.291	5.340	2.597	2.163	2.422	2.469
2022	5.715	4.759	3.209	2.727	1.781	1.745

## 4. Empirical results and analysis

### 4.1 Baseline model regression results

This study eliminates as many confounding factors affecting the results as possible, such as controlling for the influence of urban in the regression and including area and time fixed effects. Table 3 reports the regression results of Eq (1), The columns (1) and (2) of Table 3 represent the quantity of green technology innovation, while the columns (3) and (4) represent the quality of green technology innovation. From columns (1) and (2), the results show that the estimated coefficients for the low-carbon city pilot policy on the quantity of green technology innovation are all positive and significant. From columns (3) and (4), the results show that the estimated coefficients for the low-carbon city pilot policy on the quality of green technology innovation are all positive and significant. The regression results in Table 3 indicate that the low-carbon city pilot policy has a significant positive impact on both the quantity and quality of green technology innovation. Hypothesis 1 is verified: The low-carbon city pilot policy promotes urban green technology innovation.

**Table 3:** The DID method regression results.

Variables	Quantity	Quantity	Quality	Quality
	(1)	(2)	(3)	(4)
Low-carbon	0.584***	0.136***	0.625***	0.339***
	(0.057)	(0.035)	(0.071)	(0.068)
Pgdp		0.696***		0.350***
		(0.049)		(0.077)
Industrial		-0.854***		-1.042***
		(0.180)		(0.319)
Open		0.040***		-0.051***
		(0.008)		(0.012)
Fiscal		0.180***		0.081***
		(0.020)		(0.017)
Human		0.229***		0.159***
		(0.008)		(0.011)
Province-fixed effect	Yes	Yes	Yes	Yes
Year-fixed effect	Yes	Yes	Yes	Yes
Observations	5048	5048	5048	5048
R2	0.628	0.842	0.150	0.224

* p<0.1

** p<0.05

*** p<0.01.

### 4.2 Robustness test

The replacement of the green technology innovation measuring index. The quantity of green technology innovation is measured by the total number of authorized green patents, which includes the number of authorized green inventions and authorized green utility models in a given year. This indicator is a positive measure, where a higher value indicates a greater quantity of green technology innovation, and vice versa. The quality of green technology innovation is measured by the proportion of authorized green inventions to the total number of authorized patents (authorized inventions, authorized utility models and authorized appearance designs) in a given year. This indicator expressed by logarithm of total number adds 1 is a positive measure, where a higher value indicates higher quality of green technology innovation, and vice versa. The columns (1) and (2) of Table 4 represent the quantity of green technology innovation, while the columns (3) and (4) represent the quality of green technology innovation. From columns (1) and (2), the results show that the estimated coefficients for the low-carbon city pilot policy on the quantity of green technology innovation are all positive and significant. From columns (3) and (4), the results show that the estimated coefficients for the low-carbon city pilot policy on the quality of green technology innovation are all positive and significant. The regression results, as shown in Table 4, show that the quantity and quality of green technology innovation as determined by authorized patents are both significantly improved by the low-carbon city pilot policy. The benchmark regression results are robust.

**Table 4:** Robustness test results.

Variables	Quantity	Quantity	Quality	Quality
	(1)	(2)	(3)	(4)
Low-carbon	0.557***	0.121***	0.309***	0.151***
	(0.054)	(0.034)	(0.049)	(0.050)
Pgdp		0.605***		0.049
		(0.052)		(0.049)
Industrial		-0.699***		0.301
		(0.191)		(0.225)
Open		0.031***		-0.012*
		(0.008)		(0.007)
Fiscal		0.180***		0.016*
		(0.016)		(0.009)
Human		0.228***		0.113***
		(0.008)		(0.007)
Province-fixed effect	Yes	Yes	Yes	Yes
Year-fixed effect	Yes	Yes	Yes	Yes
Observations	5048	5048	5048	5048
R2	0.656	0.848	0.121	0.172

* p<0.1

** p<0.05

*** p<0.01.

### 4.3 Parallel trend test

This study employs the DID method to analyze the impact of low-carbon city pilot policy. The method requires that the treatment group and control group should have parallel variation trends in terms of green technology innovation before the implementation of low-carbon pilot city policy. Specifically, it assumes that green technology innovation between pilot cities and non-pilot cities are parallel before the implementation of low-carbon city pilot policy, and that the parallel trends are disrupted after the implementation of low-carbon city pilot policy. This study tests the parallel trend hypothesis by drawing on the research method of existing studies (Moser and Voena, 2012, Jiar, 2014) [45,46], and the model is set as follows:

$$y_{it}=\alpha +\mu_{i}+\lambda_{t}+\sum_{\tau =1}^{m}\theta_{-\tau }D_{i,t-\tau }+\theta D_{it}+\sum_{\tau =1}^{q}\theta_{+\tau }D_{i,t+\tau }+\delta X_{it}+\varepsilon_{it}$$ (2)

In Eq (2), θ-τ represents the influence in the τ period before treatment, and θ+τ represents the influence in the τ period after treatment. θ represents the influence in the current period of treatment. If the coefficient of θ-τ is not significant and the coefficient of θ+τ is significant, it indicates that the parallel trend test is passed. θ-τ takes five years before the low-carbon city pilot policy is carried out, and θ+τ takes five years after the low-carbon city pilot policy is carried out. Table 5 test results show that all five years coefficients of the quantity and the quality of green technology innovation are not significant before the implementation of low-carbon city pilot policy, and all four years coefficients of the quantity and the quality of green technology innovation are significant after the implementation of low-carbon city pilot policy. Therefore, the parallel trend hypothesis is reasonable.

**Table 5:** Parallel trend hypothesis test results.

Low-carbon	Applied Patents		Authorized Patents	
	Quantity	Quality	Quantity	Quality
	(1)	(2)	(3)	(4)
Before policy implementation fifth year	0.079	-0.093	0.032	0.074
	(0.064)	(0.112)	(0.061)	(0.080)
Before policy implementation fourth year	0.062	0.021	0.083	0.062
	(0.064)	(0.113)	(0.064)	(0.075)
Before policy implementation third year	0.096	0.053	0.051	0.022
	(0.060)	(0.115)	(0.063)	(0.065)
Before policy implementation second year	0.080	0.150	0.079	-0.008
	(0.059)	(0.105)	(0.063)	(0.058)
After policy implementation second year	0.168*	0.400**	0.162*	0.355**
	(0.088)	(0.187)	(0.087)	(0.168)
After policy implementation third year	0.200**	0.377*	0.163**	0.175**
	(0.083)	(0.195)	(0.080)	(0.089)
After policy implementation fourth year	0.147*	0.262*	0.139*	0.256**
	(0.087)	(0.147)	(0.084)	(0.124)
After policy implementation fifth year	0.171**	0.330**	0.135	0.089
	(0.085)	(0.150)	(0.087)	(0.125)
control variables	Yes	Yes	Yes	Yes
Province-fixed effect	Yes	Yes	Yes	Yes
Year-fixed effect	Yes	Yes	Yes	Yes
Observations	5048	5048	5048	5048
R2	0.844	0.233	0.850	0.184

* p<0.1

** p<0.05

*** p<0.01.

### 4.4 Placebo effect test

Another important test for assessing policy effects using the DID method is placebo effect test. Since placebo effect test results are very sensitive to pilot policy time, this study advances low-carbon city pilot policy by 3 years and 4 years. If the policy is implemented 3 years in advance, this study deletes data for 2020–2022. If the policy is implemented 4 years in advance, this study deletes data for 2019–2022. Whether 3 or 4 years in advance, data from years after the implementation of low-carbon pilot policy are deleted, to eliminate data interference placebo effect test.

Table 6 test results show that the coefficient of core explanatory variable is not significant, indicating that, all low-carbon city pilots 3 years in advance as dummy low-carbon city pilot policy time, the green technology innovation in the treatment and control group shows no different development trend. Therefore, based on the above test, the growth of urban green technology innovation does come from the low-carbon city pilot policy.

**Table 6:** Placebo effect test: Low-carbon city pilot policy time is 3 years ahead of actual implementation time.

Variables	Applied Patents		Authorized Patents	
	Quantity	Quality	Quantity	Quality
	(1)	(2)	(3)	(4)
Low-carbon	0.082	0.146	0.065	0.018
	(0.050)	(0.092)	(0.050)	(0.061)
Pgdp	0.591***	0.361***	0.464***	0.024
	(0.055)	(0.103)	(0.059)	(0.066)
Industrial	-0.621***	-1.003**	-0.406*	0.793**
	(0.216)	(0.440)	(0.227)	(0.329)
Open	0.035***	-0.059***	0.027***	-0.013*
	(0.009)	(0.014)	(0.009)	(0.008)
Fiscal	0.202***	0.053*	0.203***	-0.029**
	(0.028)	(0.028)	(0.025)	(0.014)
Human	0.255***	0.162***	0.258***	0.142***
	(0.011)	(0.017)	(0.011)	(0.011)
Province-fixed effect	Yes	Yes	Yes	Yes
Year-fixed effect	Yes	Yes	Yes	Yes
Observations	3321	3321	3321	3321
R2	0.811	0.185	0.807	0.200

* p<0.1

** p<0.05

*** p<0.01.

Table 7 test results show that the coefficient of core explanatory variable is still not significant, indicating that, all low-carbon city pilots 4 years in advance as dummy low-carbon city pilot policy time, the green technology innovation in the treatment and control group shows no different development trend, the conclusion that low-carbon city pilot policy promotes urban green technology innovation is still robust.

**Table 7:** Placebo effect test: Low-carbon city pilot policy time is 4 years ahead of actual implementation time.

Variables	Applied Patents		Authorized Patents	
	Quantity	Quality	Quantity	Quality
	(1)	(2)	(3)	(4)
Low-carbon	0.071	0.128	0.076	-0.014
	(0.046)	(0.085)	(0.046)	(0.056)
Pgdp	0.597***	0.367***	0.472***	0.013
	(0.057)	(0.105)	(0.060)	(0.068)
Industrial	-0.669***	-1.126**	-0.500**	0.793**
	(0.222)	(0.442)	(0.233)	(0.346)
Open	0.036***	-0.063***	0.029***	-0.013*
	(0.010)	(0.015)	(0.009)	(0.008)
Fiscal	0.209***	0.048	0.207***	-0.034**
	(0.031)	(0.030)	(0.027)	(0.015)
Human	0.255***	0.168***	0.258***	0.146***
	(0.011)	(0.017)	(0.011)	(0.011)
Province-fixed effect	Yes	Yes	Yes	Yes
Year-fixed effect	Yes	Yes	Yes	Yes
Observations	3152	3152	3152	3152
R2	0.807	0.197	0.804	0.202

* p<0.1

** p<0.05

*** p<0.01.

## 5. Analysis of intermediary mechanisms

### 5.1 Intermediary model construction

To verify the validity of intermediary mechanisms, this study constructs a mechanism effect model based on the baseline model (1), in which the impact of low-carbon city pilot policy on green technology innovation is significantly positive:

$$Med_{it}=\gamma +\gamma_{1}D_{it}+\gamma_{2}X_{it}+\mu_{1i}+\upsilon_{1t}+\varepsilon_{it}$$ (3)

$$Y_{it}=\phi +\phi_{1}D_{it}+\phi_{2}Med_{it}+\phi_{3}X_{it}+\mu_{2i}+\upsilon_{2t}+\varepsilon_{it}$$ (4)

In Eqs (3) and (4), Med_it_ represents mechanism variables. The mechanism variables selected in this study include: Government green input and Public engagement. Government green input (Government), expressed by logarithm of government environmental protection expenditure. This indicator is a positive measure, where a higher value indicates a higher government green input, and vice versa. Public engagement (Public), measured by total number of recommendation and proposal. This indicator is a positive measure, where a higher value indicates a higher public engagement, and vice versa. This study eliminates as many confounding factors affecting the results as possible, such as controlling for the influence of urban in the regression and including area and time fixed effects. The control variables X_it_ include economic development, industrial structure, degree of openness, fiscal support and human capital.

According to the basic idea of causal stepwise regression method, model (3) and model (4) are estimated based on the significant positive impact of low-carbon city pilot policy on green technology innovation in the baseline model (1). If the estimated coefficients for both the low-carbon city pilot policy in model (3) and the low-carbon city pilot policy and mechanism variables in model (4) are significantly positive, they indicate that the low-carbon city pilot policy can influence green technology innovation through mechanism variables, and mechanism variables play a partial mediating role. If the estimated coefficient for the low-carbon city pilot policy in model (4) is not significant, while the estimated coefficient for mechanism variables are significantly positive, they indicate that mechanism variables have a complete mediating effect.

### 5.2 Intermediary mechanisms analysis

The results of government green input intermediary mechanism are shown in Table 8. The columns (1) and (2) of Table 8 represent the quantity of green technology innovation, while the columns (3) and (4) represent the quality of green technology innovation. From columns (1) and (2), the results show that the estimated coefficients for the low-carbon city pilot policy on government green input, government green input on the quantity of green technology innovation, and the low-carbon city pilot policy on the quantity of green technology innovation are all positive and significant. This suggests that the low-carbon city pilot policy significantly increases government green input, and the government green input plays a partial mediating role in promoting the quantity of green technology innovation. From columns (3) and (4), the results show that the estimated coefficients for the low-carbon city pilot policy on government green input, government green input on the quality of green technology innovation, and the low-carbon city pilot policy on the quality of green technology innovation are all positive and significant. This indicates that the low-carbon city pilot policy can increase government green input, and government green input plays a partial mediating role in promoting the quality of green technology innovation. Hypothesis 2 is verified: The low-carbon city pilot policy promotes urban green technology innovation through government green input.

**Table 8:** Results of government green input intermediary mechanism.

Variables	Quantity		Quality	
	(1)	(2)	(3)	(4)
Low-carbon	0.161***	0.129***	0.161***	0.323***
	(0.020)	(0.035)	(0.020)	(0.069)
Government		0.041*		0.102**
		(0.021)		(0.047)
Pgdp	0.072**	0.693***	0.072**	0.343***
	(0.028)	(0.049)	(0.028)	(0.077)
Industrial	-0.380***	-0.838***	-0.380***	-1.003***
	(0.114)	(0.180)	(0.114)	(0.321)
Open	-0.050***	0.042***	-0.050***	-0.046***
	(0.007)	(0.008)	(0.007)	(0.013)
Fiscal	0.052***	0.178***	0.052***	0.076***
	(0.008)	(0.020)	(0.008)	(0.017)
Human	-0.016***	0.229***	-0.016***	0.161***
	(0.004)	(0.008)	(0.004)	(0.011)
Province-fixed effect	Yes	Yes	Yes	Yes
Year-fixed effect	Yes	Yes	Yes	Yes
Observations	5048	5048	5048	5048
R2	0.834	0.842	0.834	0.225

* p<0.1

** p<0.05

*** p<0.01.

The results of public engagement intermediary mechanism are shown in Table 9. The columns (1) and (2) of Table 9 represent the quantity of green technology innovation, while the columns (3) and (4) represent the quality of green technology innovation. From columns (1) and (2), the results show that the estimated coefficients for the low-carbon city pilot policy on public engagement, public engagement on the quantity of green technology innovation, and the low-carbon city pilot policy on the quantity of green technology innovation are all positive and significant. This suggests that the low-carbon city pilot policy significantly increases public engagement, and public engagement plays a partial mediating role in promoting the quantity of green technology innovation. From columns (3) and (4), the results show that the estimated coefficients for the low-carbon city pilot policy on public engagement, public engagement on the quality of green technology innovation, and the low-carbon city pilot policy on the quality of green technology innovation are all positive and significant. This indicates that the low-carbon city pilot policy can increase public engagement, and public engagement plays a partial mediating role in promoting the quality of green technology innovation. Hypothesis 3 is verified: The low-carbon city pilot policy promotes urban green technology innovation through public engagement.

**Table 9:** Results of public engagement intermediary mechanism.

Variables	Quantity		Quality	
	(1)	(2)	(3)	(4)
Low-carbon	0.029*	0.134***	0.029*	0.333***
	(0.015)	(0.035)	(0.015)	(0.068)
Public		0.057*		0.231***
		(0.034)		(0.064)
Pgdp	-0.014	0.696***	-0.014	0.354***
	(0.012)	(0.049)	(0.012)	(0.077)
Industrial	0.111**	-0.860***	0.111**	-1.067***
	(0.048)	(0.180)	(0.048)	(0.320)
Open	-0.013***	0.041***	-0.013***	-0.048***
	(0.003)	(0.008)	(0.003)	(0.012)
Fiscal	0.019***	0.179***	0.019***	0.076***
	(0.005)	(0.020)	(0.005)	(0.017)
Human	-0.002	0.229***	-0.002	0.160***
	(0.002)	(0.008)	(0.002)	(0.011)
Province-fixed effect	Yes	Yes	Yes	Yes
Year-fixed effect	Yes	Yes	Yes	Yes
Observations	5048	5048	5048	5048
R2	0.614	0.842	0.614	0.225

* p<0.1

** p<0.05

*** p<0.01.

## 6. Analysis of moderated mechanism

### 6.1 Moderated model construction

The Moderated model is utilized to test the impact of low-carbon city pilot policy on urban green technology innovation under the regulation of new infrastructure, with the model taking the specific form:

$$Y_{it}=\alpha +\omega_{1}D_{it}+\omega_{2}F_{it}+\omega_{3}D_{it}\times F_{it}+\delta X_{it}+\mu_{i}+\upsilon_{t}+\varepsilon_{it}$$ (5)

In Eq (5), i represents city, i = 1,2,…,285. t represents year, t = 2005,2006,… . .2022. the explained variable Y_it_, represents green technology innovation in the “i” city, in the “t” year. the explanatory variable D_it_ represents the low-carbon city pilot policy. F_it_ (Digital) represents new infrastructure, the government plays a significant role in providing financial support for new infrastructure at the current stage. Therefore, the frequency of keywords related to digital economy policy in the government work reports is used to calculate this indicator. This indicator is a positive measure, where a higher value indicates a higher new infrastructure, and vice versa. The moderated variable D_it_ *F_it_ represents the new infrastructure regulating the low-carbon city pilot policy, the coefficient of ω3 is significantly positive, which indicates that the moderated effect has a significant positive impact on the green technology innovation, and on the other hand, ω3 is significantly negative, which means that the moderated effect has a significant negative impact on the green technology innovation. This study eliminates as many confounding factors affecting the results as possible, such as controlling for the influence of urban in the regression and including area and time fixed effects. The control variables X_it_ represent the city-level control variables that change over time, including economic development, industrial structure, degree of openness, fiscal support and human capital.

### 6.2 Moderated mechanism analysis

The results of new infrastructure moderated mechanism are shown in Table 10. The column (1) of Table 10 represents the quantity of green technology innovation. From column (1), the results show that the estimated coefficients for the low-carbon city pilot policy, the new infrastructure, and moderated variable on the quantity of green technology innovation are all positive and significant. This suggests that the low-carbon city pilot policy significantly increases the quantity of green technology innovation, the new infrastructure significantly increases the quantity of green technology innovation, and the moderated mechanism significantly increases the quantity of green technology innovation. The column (2) of Table 10 represents the quality of green technology innovation. From column (2), the results show that the estimated coefficients for the low-carbon city pilot policy on the quality of green technology innovation is positive and significant, new infrastructure, and moderated variable on the quality of green technology innovation are positive but not significant. Hypothesis 4 is verified: New infrastructure enhances the impact of low-carbon city pilot policy on quantity of green technology innovation.

**Table 10:** Results of moderated mechanism.

Variables	Quantity	Quality
	(1)	(2)
Low-carbon	0.105***	0.337***
	(0.037)	(0.081)
New infrastructure	0.294***	0.066
	(0.093)	(0.153)
Low-carbon* New infrastructure	0.179*	0.003
	(0.108)	(0.212)
Pgdp	0.685***	0.349***
	(0.048)	(0.077)
Industrial	-0.819***	-1.036***
	(0.179)	(0.320)
Open	0.042***	-0.051***
	(0.008)	(0.012)
Fiscal	0.168***	0.079***
	(0.019)	(0.018)
Human	0.226***	0.159***
	(0.008)	(0.011)
Province-fixed effect	Yes	Yes
Year-fixed effect	Yes	Yes
Observations	5048	5048
R2	0.843	0.224

* p<0.1

** p<0.05

*** p<0.01.

## 7. Heterogeneity analysis

According to the previous theoretical analysis, This study divides the sample cities into two regions: the Yellow River Basin and the Yangtze River Economic Belt. For the Yellow River Basin, referring to the research of existing scholars [47], specifically including 97 cities in 9 provinces and municipalities. For the Yangtze River Economic Belt, referring to the research of existing scholars [48], specifically including 107 cities in 11 provinces and municipalities. Table 11 shows regression results for the the Yellow River Basin and the Yangtze River Economic Belt. The columns (1) and (3) represent the quantity of green technology innovation, while the columns (2) and (4) represent the quality of green technology innovation. The findings are placed under the columns (1) and (3). For the Yellow River Basin, the coefficient of low-carbon city pilot policy on green technology innovation is 0.466, indicating it surpasses the significance test at a 1% level. For the Yangtze River Economic Belt, the coefficient estimate of low-carbon city pilot policy on green technology innovation is 0.082, indicating it surpasses the significance test at a 10% level. Therefore, in terms of the degree and significance of influence, compared with the Yangtze River Economic Belt, the low-carbon city pilot policy has a higher impact on quantity of green technology innovation in the Yellow River Basin. Meanwhile, the findings are placed under the columns (2) and (4). For the Yellow River Basin, the coefficient of low-carbon city pilot policy on green technology innovation is 0.756, indicating it surpasses the significance test at a 1% level. For the Yangtze River Economic Belt, the coefficient estimate of low-carbon city pilot policy on green technology innovation is 0.204, indicating it surpasses the significance test at a 5% level. Therefore, in terms of the degree and significance of influence, compared with the Yangtze River Economic Belt, the low-carbon city pilot policy has a higher impact on quality of green technology innovation in the Yellow River Basin. Hypothesis 5 is verified: Compared with the Yangtze River Economic Belt, the low-carbon city policy has a greater influence on urban green technology innovation in the Yellow River Basin.

**Table 11:** Results of heterogeneity test.

Variables	Yellow River Basin		Yangtze River Economic Belt	
	Quantity	Quality	Quantity	Quality
	(1)	(2)	(3)	(4)
Low-carbon	0.466***	0.756***	0.082*	0.204**
	(0.068)	(0.205)	(0.050)	(0.086)
Pgdp	0.508***	0.238	0.623***	0.247**
	(0.056)	(0.147)	(0.074)	(0.107)
Industrial	-1.070***	-0.673	-0.037	-0.463
	(0.232)	(0.516)	(0.344)	(0.601)
Open	0.073***	-0.099***	0.077***	-0.053**
	(0.017)	(0.025)	(0.012)	(0.022)
Fiscal	0.125***	0.089*	0.160***	0.059***
	(0.036)	(0.046)	(0.014)	(0.018)
Human	0.230***	0.117***	0.193***	0.212***
	(0.010)	(0.018)	(0.010)	(0.017)
Province-fixed effect	Yes	Yes	Yes	Yes
Year-fixed effect	Yes	Yes	Yes	Yes
Observations	1738	1738	1908	1908
R2	0.843	0.108	0.872	0.277

* p<0.1

** p<0.05

*** p<0.01.

## 8. Conclusions and recommendations

Based on the panel data of 285 cities in China from 2005 to 2022, this study utilizes the DID method to identify the impact of low-carbon city policy on urban green technology innovation and finds the following four main conclusions: (1) The low-carbon city pilot policy promotes urban green technology innovation. (2) The low-carbon city pilot policy promotes urban green technology innovation through government green input and public engagement. (3) New infrastructure enhances the impact of low-carbon city pilot policy on quantity of green technology innovation. (4) Compared with the Yangtze River Economic Belt, the low-carbon city policy has a greater influence on urban green technology innovation in the Yellow River Basin.

Based on the above conclusions, this paper puts forward corresponding recommendations: First, Expand the experience of low-carbon city policy according to local conditions. The pilot low-carbon city policy is soft constraint environmental policy. Given the characteristics, pilot cities can formulate low-carbon policies that suit their economic development and promote urban green technology innovation. Second, Improve the government and public participation in low-carbon urban governance. Pilot cities pay attention to low-carbon governance in fiscal expenditures, alleviate the cost problem faced by green technology innovation through the government’s environmental protection expenditure, and stimulate the vitality of the cities to maintain green technology innovation through the public scrutiny. Third, Promote the construction of new infrastructure, establish a new type of infrastructure operation mechanism in line with the requirement of market economy, and enhance urban green technology innovation. Four, Implement differentiated regional development strategies. The cities of Yellow River Basin attache importance to the convergence of talents and knowledge, forming human capital spillover effect and knowledge spillover effect. The cities of Yangtze River Economic Belt focus on the quality of green technology innovation through the accumulation of knowledge and technology.

## Supporting information

S1 Data (XLS)
